# PQ-FundusChain: a postquantum blockchain framework for secure and privacy-preserving retinal fundus image sharing in teleophthalmology

**DOI:** 10.3389/fdgth.2026.1933562

**Published:** 2026-09-07

**Authors:** M. Kirubakaran, V. Vijayarajan

**Affiliations:** School of Computer Science and Engineering, Vellore Institute of Technology, Vellore, India

**Keywords:** access control, blockchain, diabetic retinopathy, fundus images, medical image security, ML-DSA, ML-KEM, postquantum cryptography

## Abstract

**Introduction:**

Teleophthalmology depends on secure exchange of retinal fundus images between primary care screening locations and referral centers. Current image-sharing systems remain vulnerable to quantum attacks on public-key cryptography and to centralized storage failures with limited auditability.

**Methods:**

This paper presents PQ-FundusChain, a consortium blockchain framework for quantum-resistant fundus image sharing. Each image is encrypted with an AES-256-GCM session key encapsulated using ML-KEM (FIPS 203). ML-DSA (FIPS 204) authenticates blockchain transactions. Encrypted images are stored in decentralized off-chain storage, while content hashes, metadata, and access policies are recorded on-chain. Access requests are evaluated through a risk-aware policy combining trust, access-history, and location scores with a fundus sensitivity score based on retinal anatomy and diabetic retinopathy severity.

**Results:**

Experimental evaluation on the public Messidor-2 dataset shows a share-side cryptographic and hashing cost of approximately 4.02 ms per image, an access-side cost of approximately 3.48 ms, smart-contract execution of approximately 0.25 ms for both AccessGrant and PolicyUpdate, and a per-image on-chain record of approximately 4.83 KB independent of image resolution. The additional latency introduced by postquantum mechanisms remains below 1 ms for image sharing.

**Discussion:**

The results demonstrate that postquantum protection can be incorporated into teleophthalmology workflows with modest computational overhead, with security analysis confirming confidentiality, integrity, authentication, non-repudiation, fine-grained access control, and resistance to harvest-now, decrypt-later attacks.

## 1 Introduction

Diabetic retinopathy (DR) is a leading cause of preventable blindness, and the global burden continues to grow: the number of adults with diabetic retinopathy is projected to rise from approximately 103 million in 2020 to roughly 160 million by (1). Population-scale screening increasingly depends on teleophthalmology, in which fundus photographs captured at primary-care sites are transmitted to referral centers for grading by specialists or automated systems (2–5).

**Figure 1:**
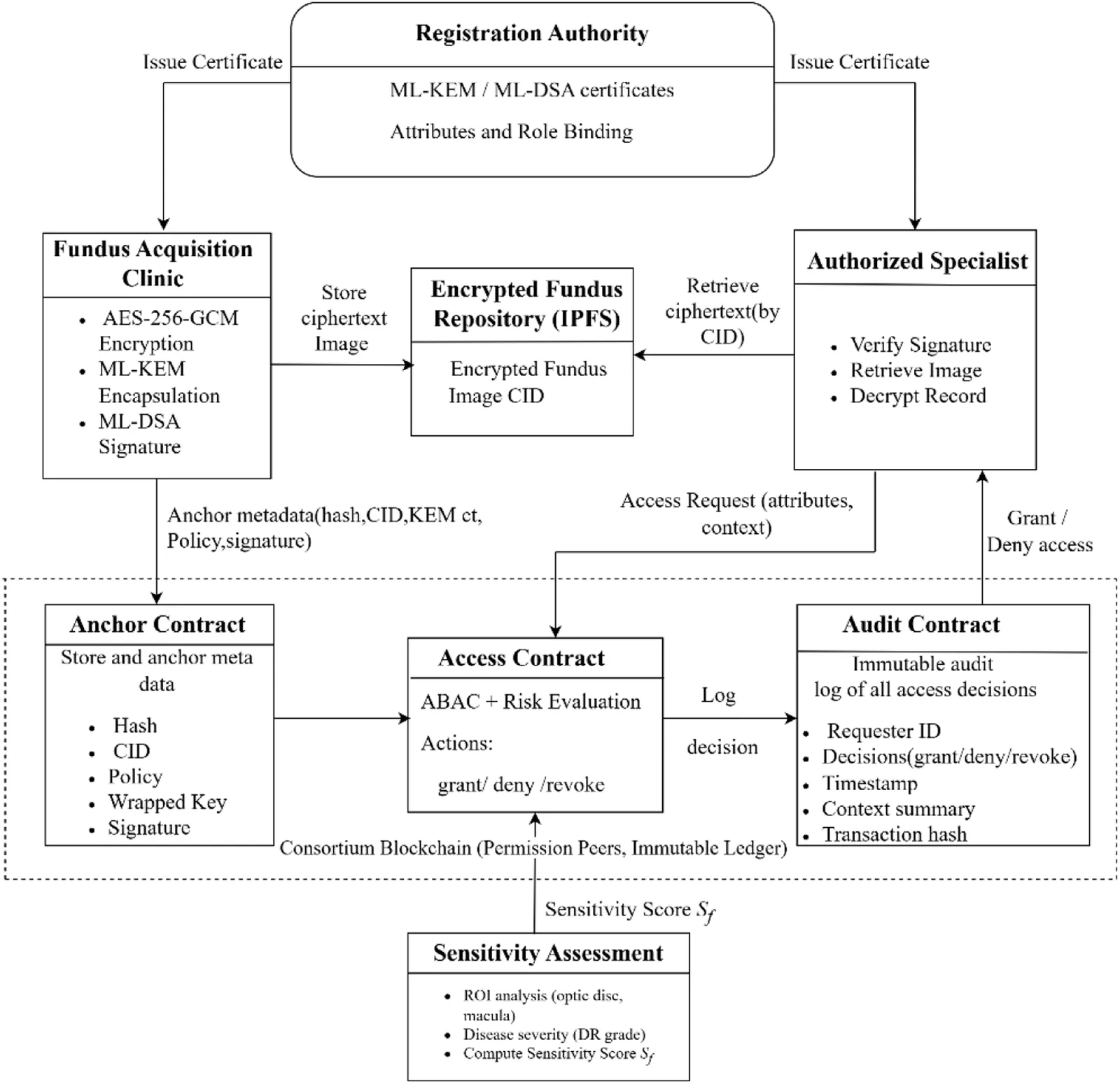
PQ-FundusChain architecture and workflow across identity, data, and control planes.

As screening volumes increase, large numbers of retinal images are exchanged between geographically distributed screening clinics, partner hospitals, and cloud or picture-archiving services. Secure image transmission, patient privacy, and regulatory compliance become core operational requirements. Fundus images constitute protected health information that is biometric in nature and linkable to a patient record, and they are subject to regulations such as the HIPAA Security Rule (6) and the General Data Protection Regulation (GDPR) (7). Because retinal images often remain clinically relevant for years and support longitudinal monitoring of disease progression, their confidentiality must hold over a long horizon. In practice, images cross hospital wireless networks, public internet links, and third-party cloud or picture-archiving systems, exposing them to interception, unauthorized access, and tampering.

Confidentiality currently relies on transport-layer and storage encryption built on RSA and elliptic-curve key establishment. Shor's algorithm (8) can break both on a large enough quantum computer. An adversary can capture ciphertext now and decrypt it later, once such hardware exists. In August 2024, the U.S. National Institute of Standards and Technology finalized the first postquantum cryptography standards: FIPS 203 [Module-Lattice Key-Encapsulation Mechanism (ML-KEM)], FIPS 204 [Module-Lattice Digital Signature Algorithm (ML-DSA)], and FIPS 205 (SLH-DSA) (9–11) and urged immediate migration to these standards (12). A fundus-image sharing framework built for long-term clinical deployment must provide cryptographic resilience against both contemporary and future adversaries. Large-scale quantum computing threatens the security assumptions behind RSA and elliptic-curve cryptography, the public-key cryptosystems most teleophthalmology platforms rely on today. That leaves stored encrypted medical data exposed to harvest-now, decrypt-later attacks. Security architectures for teleophthalmology should build in postquantum cryptographic mechanisms at the design stage and not add them later as a retrofit. A second problem has nothing to do with cryptography. Most currently used sharing infrastructures depend on centralized cloud services or trusted third parties. This arrangement results in a single point of failure, reduces transparency, and centralizes administrative control in one place. Such architecture offers limited support for independently verifiable auditability, fine-grained authorization enforcement, and immutable provenance tracking across multiple healthcare institutions. Blockchain (13)-based architectures address several of these limitations by ensuring trust across participating institutions and maintain an immutable record of every data-management event. Permissioned platforms such as Hyperledger Fabric (14) offer tamper-evident provenance, decentralized trust across cooperating institutions, machine-enforced access policies through smart contracts, and an immutable audit trail of every access decision. Storing large images directly on-chain is infeasible, and therefore, encrypted images are held off-chain in decentralized storage such as InterPlanetary File System (IPFS) (15), while the ledger anchors their content hashes and policies.

This work integrates postquantum cryptography, permissioned blockchain infrastructure, decentralized storage, and risk-aware access control into a unified framework for secure retinal fundus image sharing. Specifically, the proposed PQ-FundusChain architecture uses postquantum cryptographic primitives to protect both image confidentiality and transaction integrity. Smart contracts enforce attribute-based authorization, consent management, key delivery, auditability, and revocation. The overall architecture of PQ-FundusChain, including the identity, data, and control planes, is illustrated in Figure 1. The main contributions of this work are summarized as follows:

- We propose a hybrid storage architecture that combines off-chain encrypted image storage with on-chain anchoring of content hashes, metadata, and access policies. This design allows scalable sharing, the history of changes to be recorded and irreversible, and data management to be audited.
- We develop a smart-contract-based authorization framework that integrates attribute-based access control (ABAC), postquantum key delivery, trust-aware decision making, sensitivity-adaptive authorization thresholds, consent management, and revocation.
- We formulate a security framework and threat model that covers both classical and quantum adversaries, including harvest-now, decrypt-later attacks. We then analyze the system with respect to confidentiality, integrity, authentication, non-repudiation, accountability, and access-control requirements.
- We evaluate the proposed framework using the public deidentified Messidor-2 dataset and report cryptographic overhead, smart-contract execution performance, and storage efficiency compared with conventional RSA/ECC-based approaches.

## 2 Related work

### 2.1 Blockchain-based sharing of medical images

Blockchain with decentralized off-chain storage has been widely studied for health data. MedRec (16) introduced ledger-managed access permissions for electronic medical records, recent systems pair a permissioned ledger with IPFS, storing encrypted records and images off-chain while anchoring hashes and policies on-chain. The encrypted records and images are stored off-chain, while hashes and policies are anchored on-chain. Patient-centric frameworks such as HealthRec-Chain (17) store sensitive reports and images on IPFS with blockchain-managed permissions, and zero-trust designs (18) combine blockchain, IPFS, and edge computing to enforce patient-controlled access through smart contracts, while reducing latency. Blockchain-based electronic medical record schemes (19, 20) enable multiparty authorization and signatures over IPFS-stored ciphertext. These works establish the off-chain storage, on-chain-anchoring pattern but rely more on classical public-key cryptography without quantum resistance, and none of them target the fundus-image teleophthalmology workflow specifically.

### 2.2 Medical image encryption and privacy

Confidentiality of medical images has been pursued through standard symmetric encryption such as AES in authenticated modes (21–23) and through chaos-based (24, 25) and region-aware (26) schemes that concentrate cryptographic effort on diagnostically important regions. Complementary privacy-preserving approaches apply learning models under privacy constraints during inference (27). These mechanisms protect the payload but do not address authenticated, auditable, multi-institution sharing or the quantum threat to the key-establishment layer. Our framework treats payload encryption as one component within a quantum-resistant sharing system and can incorporate a region-aware image cipher as the symmetric primitive.

### 2.3 Postquantum cryptography and standards

Shor's algorithm renders RSA and elliptic-curve cryptography insecure against quantum adversaries, while Grover's algorithm (28) imposes only a quadratic speed-up on symmetric search. Hence, the doubling symmetric key length restores margin. ML-KEM [from CRYSTALS-Kyber (29)] is a lattice-based key-encapsulation mechanism that replaces RSA and ECDH key establishment. ML-DSA [from CRYSTALS-Dilithium (30)] is a lattice-based signature scheme replacing RSA and ECDSA. SLH-DSA (from SPHINCS+) is a hash-based alternative. ML-KEM-768 is widely recommended as the deployment baseline, while CNSA 2.0 (31) mandates ML-KEM-1024 and ML-DSA-87 for national-security systems. These standards are the cryptographic grounds of the proposed framework.

### 2.4 Quantum-resistant blockchain in healthcare

Integrating postquantum cryptography with blockchain for healthcare is an emerging research direction. Systematic reviews of quantum, postquantum, and blockchain approaches for the Internet of Medical Things (32) highlight that most deployed blockchains still depend on quantum vulnerable signatures and that few systems combine postquantum primitives with practical healthcare workloads.

Recent studies have incorporated postquantum cryptographic mechanisms into blockchain-based electronic health record systems, while reviews of quantum-resistant blockchain security (33) have identified lattice-based and hash-based alternatives to conventional public-key cryptography. Reported limitations include the absence of standardized postquantum primitives, limited support for medical image sharing workflows, and inadequate mechanisms for fine-grained revocation and access management. Our framework differs by adopting the finalized NIST primitives end to end and by targeting the fundus-image sharing pipeline with attribute-based access control and revocation.

### 2.5 Access control for shared medical data

Attribute- and role-based access control, including ciphertext policy attribute–based encryption (34), allows data vendors to express who may decrypt under which attributes. On blockchains, smart contracts encode such policies and produce an auditable record of every grant, denial, and revocation. The proposed system adapts the attribute-based, smart-contract-enforced policies and bind them to postquantum identities issued by a registration authority, so that key delivery and revocation are governed on-chain.

Table 1 summarizes how PQ-FundusChain compares with representative blockchain-based medical image sharing frameworks across the criteria most relevant to this work.

**Table 1:** Comparison of PQ-FundusChain with representative blockchain-based medical image sharing.

Framework	Block-chain	IPFS off-chain	Postquantum	Risk-aware access	Revocation	Fundus-specific
MedRec (16)	Yes	No	No	No	Partial	No
HealthRec-Chain (17)	Yes	Yes	No	No	Yes	No
Zero-trust BC + IPFS (18)	Yes	Yes	No	Partial	Yes	No
BC-EMR schemes (17, 18)	Yes	Yes	No	No	Partial	No
PQ-blockchain EHR (33)	Yes	Partial	Yes	No	No	No
IoMT PQ review systems (32)	Yes	Partial	Partial	No	Partial	No
PQ-FundusChain	Yes	Yes	Yes	Yes	Yes	Yes

Systematic comparison with representative existing approaches is widely recognized as an important validation practice for newly proposed computational frameworks. Recent numerical and fractional-order computational modeling studies have similarly emphasized benchmarking against established methods and clearly positioning new contributions within the existing literature to demonstrate methodological advances (35, 36). Following this validation strategy, Table 1 compares PQ-FundusChain with representative blockchain-based medical image sharing frameworks across postquantum security, decentralized storage, access control, revocation, and fundus-specific capabilities.

## 3 Threat model and security requirements

### 3.1 Entities and assumptions

The system comprises five roles: A capture site at the screening clinic acquires and encrypts fundus images; a referral center and its ophthalmologists or automated graders that consume images; a registration authority that issues and binds postquantum public keys to verified clinical identities and attributes; consortium blockchain peers, operated by the participating institutions, order and validate transactions; decentralized off-chain storage holds the ciphertext. We make three trust assumptions: the registration authority is trusted for identity binding, an honest majority of consortium peers holds, and therefore, consensus integrity is preserved and the symmetric cipher and the NIST postquantum primitives are secure at their standardized parameters.

### 3.2 Adversary model

We consider an adversary with the following capabilities. It can observe and record all network traffic and all ciphertext held off-chain and on-chain. It can act as a storage provider. It may compromise a minority of consortium peers or a non-authorized endpoint. It possesses, now or in the future, a cryptographically relevant quantum computer. The adversary may pursue several attacks. It can mount harvest-now, decrypt-later attacks by archiving ciphertext for future quantum decryption. It can attempt replay and man-in-the-middle manipulation, tamper with stored images or ledger records, and seek images for which it holds no authorization, including through collusion among non-authorized parties. The adversary's goal is to recover fundus images or diagnostic content, forge provenance, or obtain unauthorized access.

### 3.3 Security requirements

- Confidentiality: Image-based content is disclosed only to authorized recipients and remains protected against quantum-capable adversaries, including archived ciphertext (resistance to harvest-now, decrypt-later attacks).
- Integrity and provenance: Any modification of an image or its metadata is detectable, and the origin and history of each image are verifiable.
- Authentication and non-repudiation: The originator of every image and transaction is authenticated and cannot later deny the action.
- Authorization and access control: Only entities satisfying the data owner's policy can obtain the decryption key, with support for consent, fine-grained grants, and immediate revocation.
- Auditability: Every access decision is recorded immutably for later inspection.
- Availability: No single server is a point of failure for storage or access mediation.

## 4 Proposed framework: PQ-FundusChain with risk-aware access control

### 4.1 Overview

A similar principle of selecting methodological components according to the characteristics of the target problem has been adopted in computational disease-modeling studies (37). Following this principle, PQ-FundusChain integrates postquantum cryptography, blockchain, adaptive authorization, and fundus-data sensitivity because each component addresses a specific security requirement of teleophthalmology rather than serving as an independent enhancement, so that long-term cryptographic resilience, secure data sharing, fine-grained authorization, and clinical data sensitivity are met within a single, coherent framework.

PQ-FundusChain separates three planes: a data plane, a control plane, and an identity plane. Keeping the image data, the permission rules, and the cryptographic identities in separate layers ensures that no single component holds both the plaintext and the authority to release it. The data plane deals with only the image payload. At the capture location, each fundus image is first encrypted using a new per-image AES-256-GCM key, and the encrypted content is then saved in decentralized off-chain storage like IPFS, which provides a content identifier based on the data. Only encrypted data leave the clinic, and the original text never gets into any shared system. Because images are big and have a very long clinical life cycle, storing them off-chain allows the ledger to remain small, while still maintaining the possibility of verifying each image by referencing its hash that is on-chain. The control plane is responsible for trust and permission. On a consortium ledger, it writes down the content hash, the encrypted metadata, the access policy, and the wrapped symmetric key material. Therefore, origin and policy are unchangeable and can be checked by anyone independently. Three smart contracts are exposed: there is one anchoring contract that records each image, a risk-aware access contract that checks and controls signed requests by releasing only the wrapped key, and an audit contract that tracks every decision made. Altogether these contracts trace the entire life cycle of an image record, starting from sharing to access and revocation, and result in an unchangeable log of each access decision. The identity plane is concerned with authenticated identity. It is run by the registration authority and it issues postquantum certificates that connect the ML-KEM and ML-DSA public keys of each participant to a confirmed clinical role and attribute set. These certified identities are what the access policies in the control plane are written against, and therefore, authorization decisions resolve to cryptographically verified participants rather than unauthenticated endpoints. Across the three planes, plaintext images never touch the ledger or any single central server. The ledger mediates trust and authorization, decentralized storage holds only ciphertext, and the registration authority governs identity, and therefore, the system has no single point at which both the data and the means to decrypt it are exposed.

### 4.2 Postquantum key management

Each participant holds two postquantum key pairs: an ML-KEM key pair for confidentiality and an ML-DSA key pair for authentication. The registration authority issues postquantum certificates binding these public keys to an identity and attribute set, signed under ML-DSA. ML-KEM-768 and ML-DSA-65 play a key role as the default Category-3 parameters, with ML-KEM-1024 and ML-DSA-87 available for deployments following CNSA 2.0. A hybrid mode that concatenates an X25519 secret with the ML-KEM secret can be enabled during migration so that confidentiality holds if either component is secure. Table 2 summarizes the standardized parameter sizes of the adopted postquantum cryptographic schemes, including public keys, secret keys, ciphertexts, and digital signatures, and illustrates that ML-KEM-768 and ML-DSA-65 achieve NIST Category-3 security at the cost of increased communication and storage overhead relative to conventional public-key cryptographic schemes.

**Table 2:** Standardized parameter sizes and NIST security categories of the adopted postquantum cryptographic schemes.

Scheme/parameter	Public key (B)	Secret key (B)	Ciphertext/signature (B)	NIST category
ML-KEM-512	800	1632	768 (ciphertext)	1
ML-KEM-768	1184	2400	1088 (ciphertext)	3
ML-KEM-1024	1568	3168	1568 (ciphertext)	5
ML-DSA-44	1312	2560	2420 (signature)	2
ML-DSA-65	1952	4032	3309 (signature)	3
ML-DSA-87	2592	4896	4627 (signature)	5

### 4.3 Image confidentiality (hybrid encryption)

For each image P, the capture site samples a fresh 256-bit symmetric key k and computes the ciphertext

$C=\mathrm{AES}-256-\mathrm{GC}M_{k}(P)$, obtaining an authentication tag *t* that protects integrity. To share with an authorized recipient holding ML-KEM public key $pk_{r}$, the site runs ML-KEM encapsulation to produce a KEM ciphertext ct and shared secret ss, derives a wrapping key from ss with a key-derivation function, and wraps *k*. The recipient later recovers ss by ML-KEM decapsulation with its secret key and unwraps *k*. Symmetric strength at the 256-bit level preserves margin against Grover's quadratic search. When a region-aware image cipher is preferred, it can replace AES-256-GCM as the symmetric primitive without changing the key-management or ledger design.

### 4.4 Off-chain storage and on-chain anchoring

The ciphertext C is written to decentralized off-chain storage, which returns a content identifier derived from the data. The capture site computes a hash digest h=SHA3−256(C) (50) and submits a transaction containing h, the content identifier, the ML-KEM ciphertext ct, encrypted metadata, the access policy, and an ML-DSA signature over these fields. Anchoring the hash on the immutable ledger makes any later modification of the stored ciphertext detectable, and the signature establishes origin and non-repudiation. Because only a hash, a content identifier, and short key material are stored on-chain, ledger growth is independent of image size.

### 4.5 Attribute-based and risk-aware access control

Two mechanisms govern access authorization: an attribute-based policy tied to each image record, and a risk-aware decision model. When the recipient requires a permission, they have to send the authorization request signed by their ML-DSA. The access-control smart contract will first verify the requester's certified attributes with the access policy. Second, it will measure a risk score to determine whether the key should be given. This two-stage procedure differs from the conventional ABAC because it also involves an adaptive risk assessment element. Therefore, the access decisions look not only at the compliance of the policy but also at the trust factors that are derived from the context. This method is consistent with the principles of ABAC as documented in NIST SP 800-162 (38), and it also supports risk-based access control models that use contextual information to make authorization decisions (39).

For a requesting user *u*, the contract estimates a composite risk score based on three normalized components, each of which is within the interval [0, 1]

$$R_{u}=\alpha T_{u}+\beta A_{u}+\gamma L_{u}\;\;,\alpha +\beta +\gamma =1$$ (1)

The overall risk score $R_{u}$, defined in Equation (1), is a weighted combination of the trust score, access-history score, and location score. Access will be granted only if the attribute-based policy is satisfied and the computed risk score is above the authorization threshold ($R_{u}$ ≥ *τ*, where *τ* ∈ (0, 1]). The trust score $T_{u}$ measures the requester's level of reliability by considering the strength of the credentials, the reputation of the institution within the consortium, and the history of compliant behavior. The access-history score $A_{u}$ helps in measuring how well the present request aligns with the known access patterns. It therefore identifies any abnormality in the frequency, time, or quantity of requests. On the other hand, the location score $L_{u}\;$ checks how strongly the context of a request, such as network origin, device characteristics, and physical location, aligns with the authorized operational profile of the requester. The weights *α*, *β*, and *γ* are policy parameters stored, with *τ*, in the image record. To ensure that the access decision can be replicated, the access-history and location scores are given functional definitions. The access-history score, represented by Equation (2), quantifies the extent to which a requester's present access behavior diverges from the expected baseline

$$A_{u}=1-\min\left(1,\frac{\left|\;f_{u}-f_{ref}\right|}{\;f_{max}}\right)$$ (2)

where $f_{u}$ denotes actual request frequency, $f_{ref}$ stands for theoretical frequency for that role, and $f_{max}$ refers to the largest permitted divergence for the purpose of normalization. $A_{u}$ is almost 1 for actions matching the usual access patterns and gradually reduces to 0 when discrepancies get bigger. Location scoring is carried out using features of the access request situation, such as network origin, device profile, and geographical location.

The location score $L_{u}\;\;\;$ is assigned based on the trustworthiness of the request origin. A score of 1.0 is assigned to requests originating from trusted consortium networks, 0.7 to recognized external networks, and 0.3 to unrecognized networks. Both scores lie in [0, 1] and feed the composite risk score together with the trust score. The parameters ($f_{ref}$, $f_{max}$), location-tier definitions, weighting coefficients, and authorization threshold *τ* are deployment-specific settings maintained by the access-control framework and calibrated using operational access logs.

#### 4.5.1 Design rationale for the risk weights

A recommended default configuration of *α* = 0.5, *β* = 0.3, *γ* = 0.2 adopted and assigning the greatest weight to the trust score, which captures long-term user reliability through exponential weighted moving average (EWMA)-based aggregation of historical behavior (40). The access-history and location scores serve as contextual indicators that complement the authorization decision, while the equal-weight configuration (*α* = *β* = *γ* = 1/3) is retained as a baseline and may be refined using operational access logs or the analytic hierarchy process (AHP) (41).

Changes in trust due to compliant, abnormal, and malicious behaviors are shown in Figure 3. Unlike fixed trust models, PQ-FundusChain changes the trust score adaptively after each interaction with the help of an EWMA (42), so that the trust score not only reflects cumulative behavioral evidence but can still be sensitive to recent activity. Equation (3) depicts the approach of PQ-FundusChain to updating trust:

$$T_{u}(t+1)=\lambda T_{u}(t)+(1-\lambda )B_{u}(t),\;\;\lambda \in [0,1]$$ (3)

where $B_{u}(t)$ in [0, 1] represents the latest interaction's behavior score (high for compliant access, low after a policy violation or integrity failure) and *λ* is a memory factor trade-off between stability and responsiveness. The updated trust score is incorporated into the overall risk assessment in Equation (4):

$$R_{u}=\alpha T_{u}(t+1)+\beta A_{u}+\gamma L_{u},\alpha +\beta +\gamma =1$$ (4)

The $R_{u}$ denotes the overall risk score, and $\left[T_{u}(t+1)\right]$ is the updated trust score. By regularly updating and recording trust scores after every authorization decision, this framework supports an adaptive, history-aware access control system in which repeated breaches of the policy gradually lower a user's trust level and authorization eligibility over time.

Permissions for accessing data may be restricted to particular receivers, roles, or time periods. Revocation is done through a smart-contract transaction that stops the key from being delivered in the future and also logs the action in the blockchain. This results in an entire, verifiable access history. User consent is afforded as a policy attribute and is checked during the authorization process. Smart-contract functions implement the risk-aware access-control mechanism.

These functions are responsible for authorization, key delivery, policy management, and revocation. The related workflows are specified in Algorithms 2 and 3, which outline the secure image-sharing process and the risk-aware access, key-delivery, and revocation procedures. Every authorization decision, policy update, and revocation event is permanently logged on the ledger.

As a worked example, with weights (*α*, *β*, *γ*) = (0.5, 0.3, 0.2) and threshold *τ* = 0.7, a routine request from a credentialed grader at a known consortium site with $T_{u}$  = 0.9, A_u  = 0.8, and $L_{u}$ = 1.0 yields $R_{u}$  = 0.89 ≥ *τ* and is granted. A request having the same qualities as a previous one but coming from an unknown source as part of an unusual burst, where $T_{u}$  = 0.6, A_u  = 0.3, and $L_{u}$  = 0.2, results in $R_{u}$  = 0.43 < *τ* and is denied, while the static attributes are identical. Therefore, the system possesses a compromised yet legitimate credential that a merely attribute-based policy would not. Permissions can be limited to certain individuals, roles, or time periods, and revocation is done by a contract call that not only aborts future key delivery but is also recorded, thus creating an auditable history. The data owner's consent is represented as an attribute condition in the policy. The contract for risk-aware access reveals two different methods. First of all, AccessGrant examines a signed request, and if it works, it not only returns the wrapped key but also raises the trust score of the requester, and the whole process that starts with signature verification, goes through policy evaluation, risk scoring, and key delivery, and ends with trust-based revocation is presented in Algorithm 3. PolicyUpdate lets the data owner revise the access policy, weights, or thresholds. Both operations are recorded in the audit log, and their measured on-chain execution cost is reported in Section 7 (Table 10).

### 4.6 Fundus-data sensitivity and adaptive security level

The clinical sensitivity of fundus images varies according to their diagnostic content. To account for this variation, a fundus-data sensitivity score ($S_{f}$) is defined to quantify the relative importance of an image based on clinically relevant retinal features and represented in Equation (5):

$$S_{f}=w_{1}R_{M}+w_{2}R_{D}+w_{3}R_{P},\;w_{1}+w_{2}+w_{3}=1$$ (5)

where $R_{M}$ is the macular information density, $R_{D}$ is the optic-disc information density, and $R_{P}$ is the pathology burden, each normalized to [0, 1]. The inclusion of the macula and optic disc is clinically motivated, as these regions contain diagnostically significant information related to diabetic retinopathy progression and retinal structure, while also serving as potential biometric identifiers (43, 44). Consequently, they represent the most clinically and privacy-sensitive regions of fundus images and therefore contribute directly to the sensitivity score.

The pathology burden $R_{P}$ reflects disease severity, assigning greater sensitivity to images exhibiting advanced diabetic retinopathy. Equal weighting ($w_{1}$= $w_{2}$ = $w_{3}$  = 1/3) is adopted as the default configuration, while alternative weighting strategies, including expert elicitation, entropy-based weighting, and the AHP, are left for future work. The resulting sensitivity score is used to adapt both the protection level and the access-control policy. Specifically, higher sensitivity scores increase the extent of region-aware encryption and tighten the authorization threshold according to

$$\tau_{eff}=\;\min(1,\tau_{0}+\;\kappa \cdot S_{f})$$ (6)

In Equation (6), *τ*₀ is a base authorization threshold and *κ* is the sensitivity scaling factor; S*_f_* is the fundus-data sensitivity score. The following condition $R_{u}$  ≥  $\tau_{eff}$ enables the authorization only when the computed risk score satisfies the decision rule.

**Figure 2:**
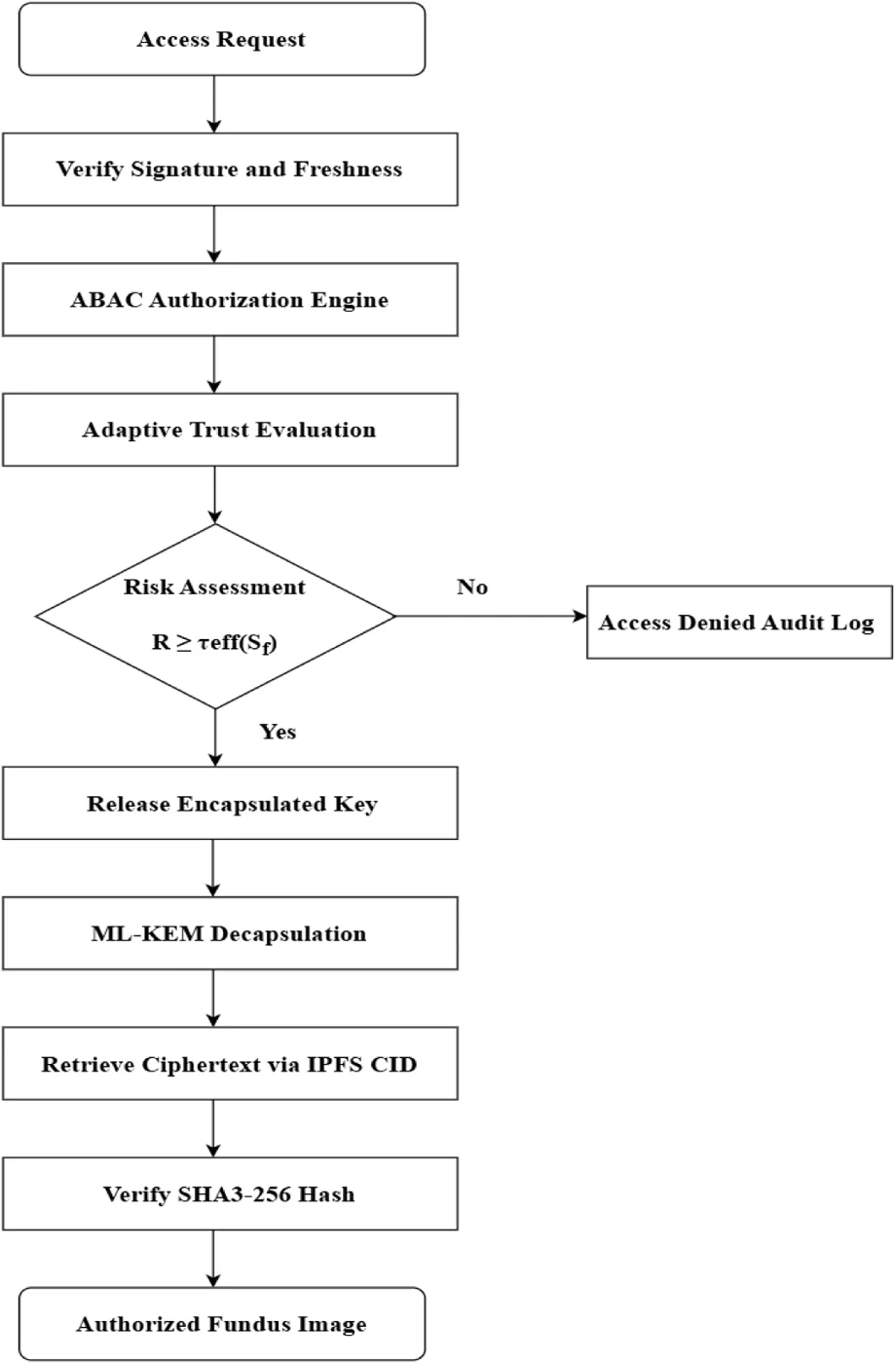
Risk-aware access control and secure image retrieval pipeline in PQ-FundusChain.

Table 3 gives an illustrative banding of $\tau_{eff}\;$ with *τ*₀ = 0.70 and *κ*=0.33. Figure 2 shows the complete access workflow, including the adaptive-trust update, the sensitivity-adjusted threshold, and the risk-aware decision block.

**Table 3:** Illustrative banding of the sensitivity-adjusted access threshold $\tau_{\mathrm{eff}}$ (*τ*₀ = 0.70, *κ*=0.33).

Sensitivity band	S*_f_* range	Effective threshold $\tau_{eff}$
Mild	0.0–0.3	0.70
Moderate	0.3–0.6	0.80
Severe	0.6–1.0	0.90

As the sensitivity score increases, the effective authorization threshold becomes progressively strict. The full mapping is plotted in Figure 5.

#### 4.6.1 Computation of the sensitivity components

To make $S_{f}$ reproducible, the three components are obtained directly from the region-of-interest analysis used for region-aware encryption: $R_{M}$ and $R_{D}$ are the macular and optic-disc Region-Of-Interest (ROI) areas expressed as fractions of the fundus area (information density), and $R_{P}$ is the diabetic-retinopathy grade normalized to the unit interval and is represented by $R_{M}=N_{M}/N_{F}$, $R_{D}=N_{D}/N_{F}$, and $R_{P}=G/4$, where $N_{M}$ represents the number of pixels within the macular region of interest, $N_{D}\;$ is the number of pixels within the optic-disc ROI, $N_{F}$ is the total number of pixels in the fundus image, and *G* represents the diabetic retinopathy grade. Table 4 reports these quantities computed on representative fundus images with equal weighting of the optic disc, and macula are localized by the morphological step described above; the grade is taken from the dataset label (Messidor-2 provides grades 0–4). On these images, the area-based terms are small and similar across cases, and therefore, the pathology burden dominates Sf, and the sensitivity-adjusted threshold $\tau_{eff}$ rises from 0.71 for a no-DR image to approximately 0.80 for a severe case, automatically demanding a higher risk score before a more clinically sensitive image is released.

**Table 4:** Computed fundus sensitivity scores for representative fundus images (equal weighting scheme).

Image (grade)	$R_{M}$	$R_{D}$	$R_{P}$	$S_{f}$	$\tau_{eff}$
No DR (grade 0)	0.099	0.014	0.00	0.038	0.712
Boundary (grade 2)	0.101	0.019	0.50	0.207	0.768
Poor quality (grade 2)	0.101	0.016	0.50	0.206	0.768
Severe (grade 3)	0.104	0.018	0.75	0.291	0.796

Representative examples are presented for illustration; the sensitivity score is computed for every image during framework operation. The workflow demonstrates how adaptive trust and image sensitivity jointly govern key-release decisions.

### 4.7 Protocols

#### 4.7.1 Enrollment

A participant generates ML-KEM and ML-DSA key pairs, proves its identity to the registration authority, and receives a postquantum certificate binding its public keys and attributes. The certificate's hash is anchored on-chain (Algorithm 1). The image is encrypted by the capture site that stores the ciphertext off-chain, wraps the symmetric key to each intended recipient, and submits a transaction anchoring the record with the access policy (Algorithm 2). When a recipient sends a signed access request, the contract matches the requester's attributes against the policy and computes the risk score $R_{u}$. Once it is confirmed that $R_{u}$  ≥ *τ*, the contract issues the wrapped key and records the decision (Algorithm 3). The recipient retrieves the ciphertext by content identifier, verifies the on-chain hash, decapsulates to recover the shared secret, unwraps k, and decrypts the ciphertext. The data owner or registration authority calls the revocation function. Subsequent access requests for the affected record or identity fail policy evaluation, and the revocation is recorded immutably.

Algorithm 1 **Enrollment and postquantum identity registration.**

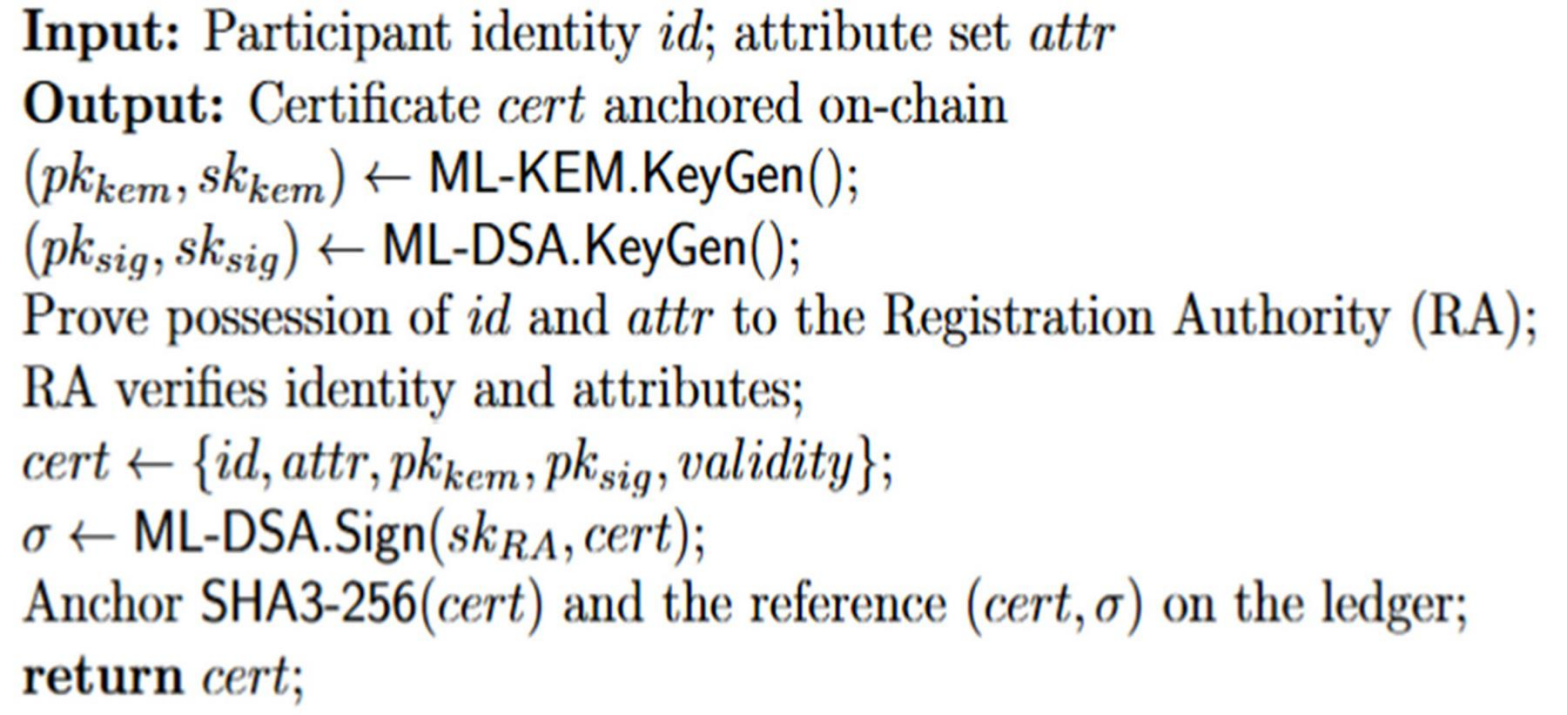

Algorithm 2 **Fundus Image encryption and secure sharing.**

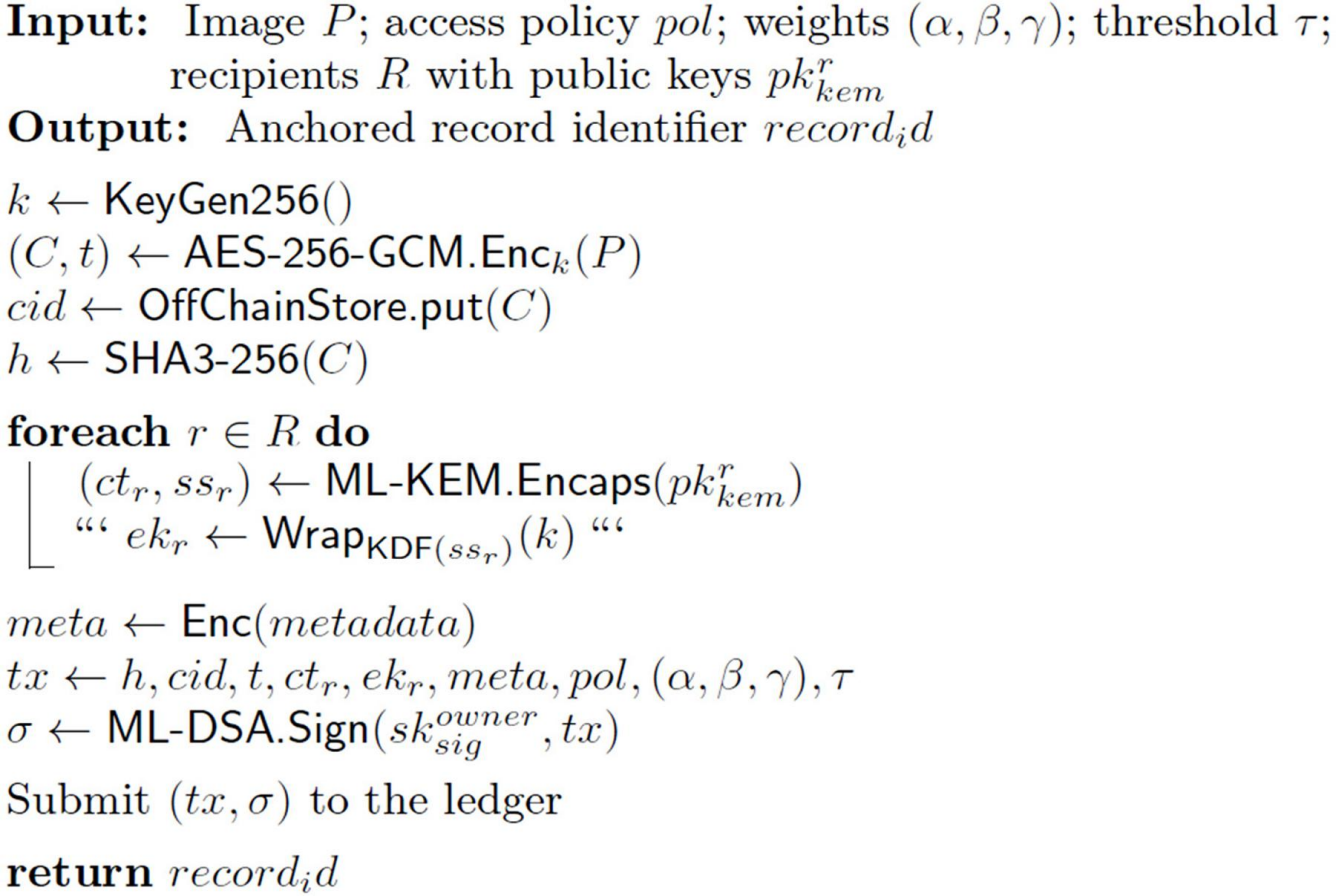

Algorithm 3 **Risk-aware access, key delivery, and revocation.**

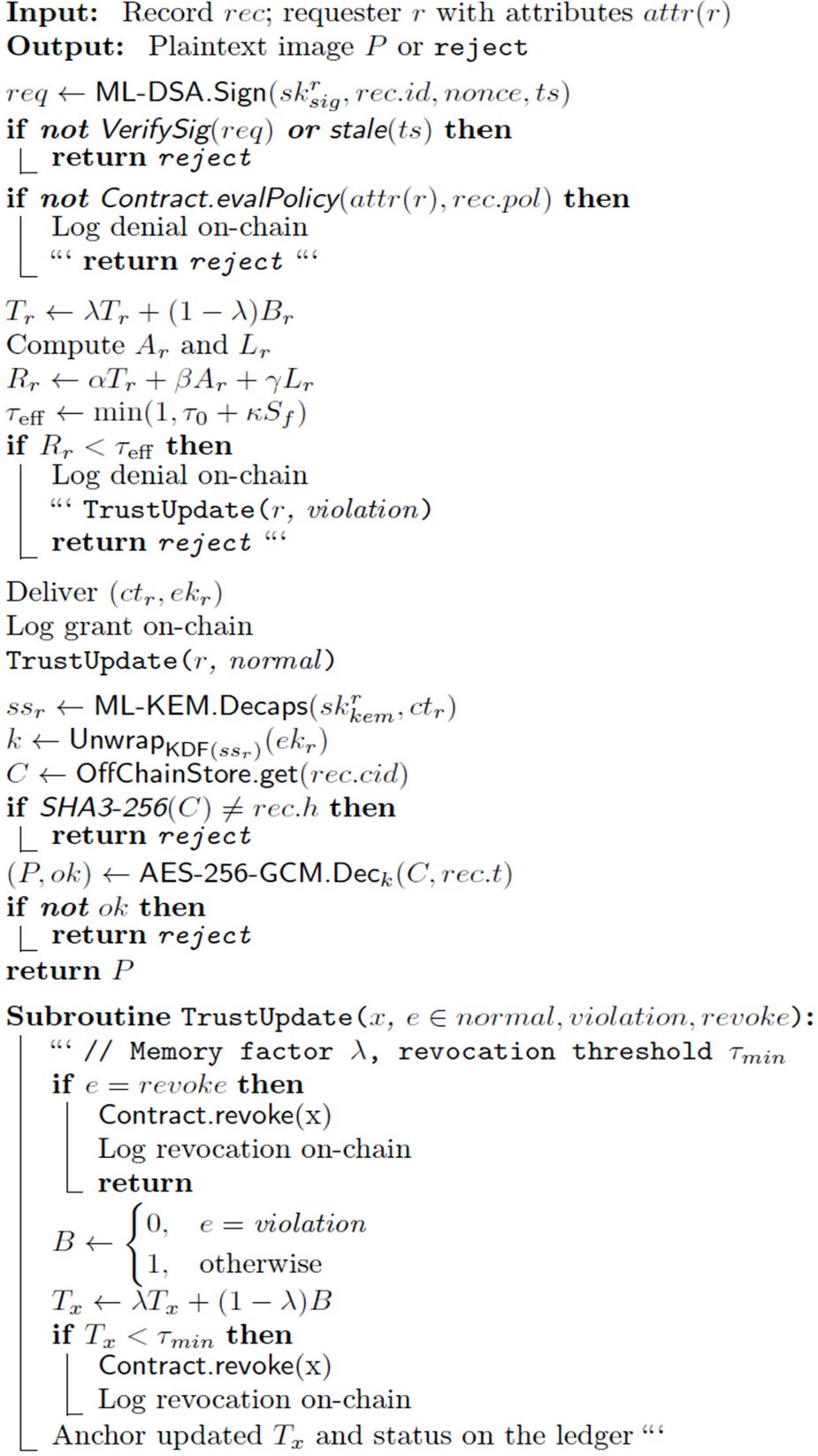

## 5 Security analysis

Table 5 maps each security objective to the concrete mechanism that provides it. The remainder of this section argues each property in turn.

**Table 5:** Security objectives and corresponding protection mechanisms.

Security objective	Supporting mechanism
Confidentiality	AES-256-GCM payload encryption with ML-KEM (FIPS 203) key encapsulation
Authentication	ML-DSA (FIPS 204) signatures over requests with fresh nonces and timestamps
Integrity	AES-GCM authentication tag and on-chain SHA3-256 content hash
Non-repudiation	ML-DSA transaction signatures recorded immutably on the ledger
Postquantum confidentiality (HNDL-resistant)	ML-KEM encapsulation and 256-bit symmetric keys (Grover margin)
Access control	Attribute-based policy with the risk-aware decision $R_{u}\geq \tau_{eff}$
Auditability	Immutable consortium ledger and audit smart contract
Revocation	Access/policy smart contract (PolicyUpdate and revocation functions)
Availability	Decentralized off-chain storage and multipeer consensus

*Confidentiality and harvest-now, decrypt-later resistance*: Plaintext is protected by AES-256-GCM, and the symmetric key is delivered only through ML-KEM encapsulation to authorized public keys. Recovering a key from archived traffic requires breaking ML-KEM, whose security reduces to the hardness of the Module Learning-With-Errors problem, believed intractable for quantum adversaries, and therefore, harvest-now, decrypt-later attacks fail. The 256-bit symmetric level retains adequate margin under Grover's algorithm. We note that ML-KEM encapsulation under long-term recipient keys provides confidentiality of archived ciphertext against future quantum decryption rather than per-session forward secrecy. An ephemeral or hybrid key-exchange mode can be layered on during migration where forward secrecy of the transport channel is required.

*Integrity, provenance, and non-repudiation*: The GCM tag detects tampering of the payload, and the on-chain SHA3-256 hash detects modification of stored ciphertext. Every transaction is ML-DSA-signed, and thus, origin is authenticated and cannot be repudiated, and the ledger's immutability preserves provenance.

### 5.1 Authentication and access control

Recipients authenticate by ML-DSA signatures over fresh nonces and timestamps, defeating replay, and man-in-the-middle alteration is detected by signature verification. Authorization is enforced by the access-control contract against certified attributes, and collusion among non-authorized parties yields no key because none satisfies the policy and none can decapsulate another's KEM ciphertext.

*Adaptive containment of compromised credentials*: The risk-aware rule adds a defense that static attribute policies lack. Because authorization requires $R_{u}$  ≥ *τ* in addition to attribute satisfaction, a credential that is valid but used anomalously, for example from an unrecognized endpoint or in an abnormal access burst, yields a low access-history or location score and is denied. The update of the trust score on-chain after violations continues to lower the standing of a misbehaving identity over time, thus minimizing damage from a partially compromised but still authorized account in a nominal sense.

**Figure 3:**
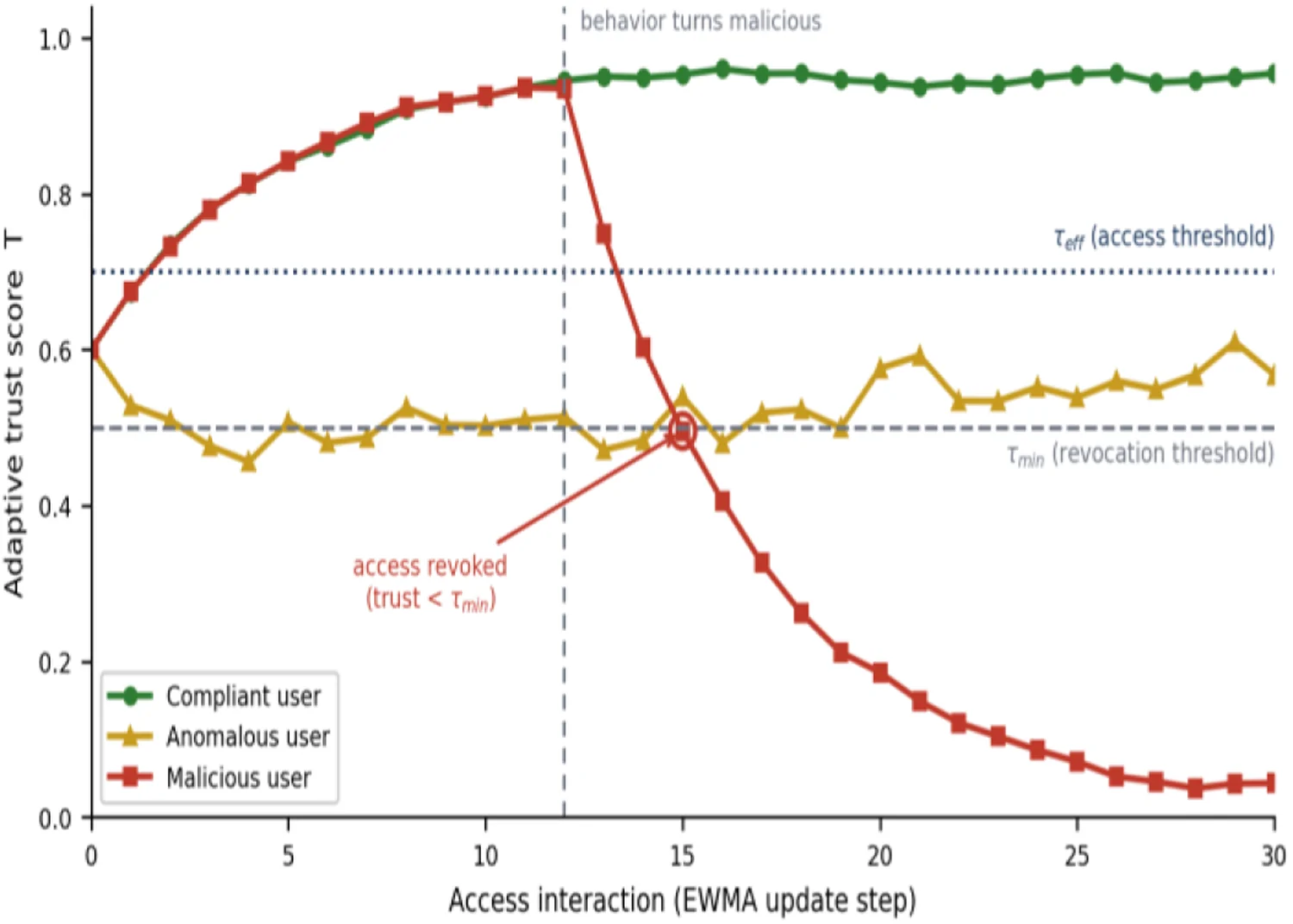
Adaptive trust monitoring and automatic access revocation mechanism.

Figure 3 was generated by simulating the trust update of Algorithm 3 with memory factor *λ* = 0.8, initial trust $T_{0}$ = 0.60, revocation threshold $\tau_{min}$ = 0.50, and access threshold $\tau_{eff}$ = 0.70 over 30 update steps. The compliant user consistently maintains high trust through positive behavior, the malicious user initially gains trust but is eventually revoked after sustained malicious actions cause its trust score to fall below $\tau_{min}$, and the anomalous user exhibits fluctuating behavior that keeps its trust score below the access threshold $\tau_{eff}$ for much of the observation period. Ledger integrity and availability are preserved through consortium consensus, decentralized storage, and multipeer validation, which prevent record tampering and eliminate the single point of failure associated with centralized servers. Formal verification of the access and key-delivery protocols using tools such as ProVerif or Tamarin is left for future work to validate key security properties, including the secrecy of *k* and the authentication of share and access messages. Table 6 summarizes how each adversary capability identified in the threat model is countered by a corresponding mechanism in PQ-FundusChain.

**Table 6:** Threat coverage of PQ-FundusChain.

Threat/capability	Mitigation mechanism
Harvest-now, decrypt-later	ML-KEM key encapsulation and 256-bit symmetric keys
Quantum forgery of identity/transaction	ML-DSA signatures (FIPS 204)
Ciphertext tampering (off-chain)	On-chain SHA3-256 anchor and AES-GCM tag
Ledger record tampering	Consortium consensus and immutable ledger
Replay/man-in-the-middle	Signed nonces and timestamps
Unauthorized access/collusion	Attribute-based smart-contract policy, per-recipient KEM
Compromised but authorized credential	Risk-aware score $R_{u}$ ≥ *τ* on-chain trust update
Central server compromise	Decentralized off-chain storage on-chain mediation
Key compromise	Revocation contract, key rotation, and per-image keys

## 6 Experimental setup

### 6.1 Implementation

The implementation choices are summarized in Table 7.

**Table 7:** Implementation summary for PQ-FundusChain.

Component	Choice/setting
Ledger	Permissioned (Hyperledger Fabric), access and anchoring as chain code
Key encapsulation	ML-KEM-768 (FIPS 203), via liboqs/Open Quantum Safe
Digital signature	ML-DSA-65 (FIPS 204)
Symmetric cipher	AES-256-GCM
Hash	SHA3-256
Off-chain storage	IPFS (content-addressed)
Classical baselines	RSA-3072, ECDSA/ECDH P-256
Agents	Python (capture/recipient) and Go (chaincode)
Hardware	Intel Xeon @ 2.80 GHz
Measurement runs	200–300 per crypto primitive and 3,000 for contract execution

Experiments were conducted on Ubuntu 22.04.5 LTS (64-bit) using Python 3.11.9, Go 1.22.5, Hyperledger Fabric 2.5.10, liboqs/Open Quantum Safe 0.12.0, GCC 13.3.0, and OpenSSL 3.0.13 (for the classical RSA/ECC baselines). A complete requirements/lockfile accompanies the released code to support independent reproduction of the measurements reported in Section 7.

### 6.2 Dataset description

The proposed framework was evaluated using the Messidor-2 dataset (45), a publicly available collection of macula-centered retinal fundus images widely used in DR research. The dataset comprises 1,748 images from 874 examinations, with paired images corresponding to the left and right eyes.

Image resolutions vary with the type of devices used for capturing the images. Considering that images are preserved in their original sizes without any alteration, the encryption, storage, and throughput measurements reflect the different file sizes that normally occur in teleophthalmology workflows quite accurately. DR severity levels are in accordance with the five-level International Clinical Diabetic Retinopathy (ICDR) scale (46, and indeed, a number of retinal experts individually graded the same images and then had a face-to-face meeting to resolve disagreements. We regard Messidor-2 as the most appropriate dataset for our research because classification performance is not the primary focus of the proposed framework, which is also the case in this research.

Actually, retinal images are regarded as protected digital assets that are used for assessment of encryption, off-chain storage, secure sharing, and adaptive access-control mechanisms. The addition of DR grade is a metadata feature that supports the pathology part of the sensitivity score that controls risk-aware access decisions, rather than being the prediction target. Currently, this research is focused only on Messidor-2. However, the framework is basically source image–independent, and other publicly available fundus datasets such as IDRiD (47 and APTOS (48) could be used instead. Hence, this work does not deal with classifier development, benchmarking, and cross-dataset generalization topics.

### 6.3 Evaluation metrics

The assessment involves four metric categories: cryptographic capabilities, the expense of hiding payload, extra on-chain storage, and the overall latency. Specifically, the measurement data were acquired in the environment of Table 7, and are reported as mean ± standard deviation of repeated runs.

*Cryptographic performance*: Execution times were measured for ML-KEM key generation, encapsulation, and decapsulation and ML-DSA key generation, signing, and verifying. In addition to the execution times, the sizes of the corresponding artifacts such as public keys, secret keys, ciphertexts, and signatures were also determined. To measure the overhead of postquantum cryptography, similar measurements were performed for RSA-3072 and ECDSA/ECDH P-256, and the results are presented in Table 8.

**Table 8:** Measured cryptographic performance of postquantum and classical security primitives.

Operation	PQ-FundusChain (ms)	Classical baseline (ms)
KEM/key-transport key generation	0.068 (ML-KEM-768)	176.6 (RSA-3072)
Key encapsulation/RSA-OAEP wrap	0.077	0.051
Key decapsulation/RSA-OAEP unwrap	0.095	1.974
Signature key generation	0.198 (ML-DSA-65)	0.020 (ECDSA P-256)
Sign (64-byte digest)	0.760	0.031
Verify	0.200	0.096
AES-256-GCM encrypt (1 MB image)	0.257	0.257
AES-256-GCM decrypt (1 MB image)	0.250	0.250
SHA3-256 hash (1 MB image)	2.93	2.93

*Payload-protection cost*: The time taken for encryption with AES-256-GCM and generating a hash with SHA3-256 was measured for each image. We employed original Messidor-2 resolutions without resizing in order to maintain the variability of file sizes as encountered in teleophthalmology workflows.

*On-chain storage overhead*: As fundus images are not saved on-chain, an analysis of storage was carried out on the ledger record that holds the content hash, wrapped key material, encrypted metadata, access policy, and transaction signature. Record size was computed from the standardized cryptographic artifacts and evaluated as the number of authorized recipients increased, with each recipient contributing one ML-KEM encapsulation. The resulting storage growth is reported in Table 9.

**Table 9:** Estimated on-chain storage overhead per fundus image.

On-chain field	PQ size (B)	Classical size (B)
SHA3-256 content hash	32	32
Off-chain content identifier (CID)	59	59
KEM ciphertext + wrapped key (per recipient)	1,088 + 40	384
Transaction signature	3,309	72
Encrypted metadata	256	256
Access policy + risk parameters	160	160
Total per image, single recipient	≈ 4,944 (≈ 4.83 KB)	≈ 963 (≈ 0.94 KB)
Each additional recipient	+1,128	+384

*End-to-end latency*: Sharing and access workflows were evaluated by separating cryptographic processing from smart-contract execution. Cryptographic latency includes key management, encryption, hashing, and signature operations, whereas ledger latency corresponds to the execution time of the AccessGrant and PolicyUpdate smart-contract functions. The results are reported separately in Table 10 to distinguish protocol overhead from blockchain processing costs.

**Table 10:** End-to-end performance metrics of the proposed framework.

End-to-end metric	PQ-FundusChain	Classical	Notes
Share-side crypto + hashing (ms)	4.02	3.27	Per image, single recipient, and excludes transfer and commit
Access-side crypto + hashing (ms)	3.48	5.25	Excludes ledger query and retrieval
Smart-contract execution: AccessGrant/PolicyUpdate (ms)	0.25/0.25 (measured)	—	On-peer chaincode compute and excludes ordering/consensus
Validation-bound throughput, single peer (tx/s)	≈ 4,000 (measured)	—	Compute only and excludes ordering, consensus, and network
Network commit latency/end-to-end transactions per second (TPS)	deployment-level (not modeled)	—	Requires a deployed multipeer Fabric testbed

Network-level metrics, including consensus latency, throughput under load, block-confirmation time, and large-scale deployment scalability, depend on consortium configuration and deployment topology, which puts them outside the scope of this non-deployed evaluation. This study instead focuses on cryptographic costs, chaincoexecution latency, and storage overhead.

## 7 Results and discussion

Comprehensive performance analysis supported by quantitative comparison with established baseline methods is a widely accepted validation strategy for newly proposed computational frameworks (49). Following this principle, PQ-FundusChain is evaluated against conventional RSA-3072 and ECDSA/ECDH P-256 mechanisms using cryptographic latency, on-chain storage overhead, smart-contract execution time, and end-to-end processing cost, to demonstrate the computational feasibility of incorporating standardized postquantum cryptography into teleophthalmology workflows.

Cryptographic operations were benchmarked with the ML-KEM-768 and ML-DSA-65 reference C implementations (PQClean, via the pqcrypto bindings) and with AES-256-GCM, SHA3-256, RSA-3072, and ECDH/ECDSA over P-256 from a standard cryptographic library, on an Intel Xeon processor at 2.80 GHz, averaging 200–300 runs per operation after warm-up. These are software reference timings on a single core, the results are implementation- and platform-dependent, and optimized implementations may achieve different performance characteristics. The symmetric-encryption and hash timings were measured on a representative 1 MB image because these per-image costs scale with file size, the corresponding Table 8 entries are per-megabyte reference values, and the native, unresized Messidor-2 images vary in size around this figure. Smart-contract execution time was measured by running the AccessGrant and PolicyUpdate logic (ML-DSA-65 verification, attribute-policy evaluation, risk-score computation, and SHA3-256 audit hashing) over 3,000 iterations after warm-up on the same processor. On-chain storage in Table 9 is computed from the standardized field sizes. Ledger throughput, commit latency, and block confirmation time depend on the consortium network and are left as deployment-level measurements, not reported here.

The measured cryptographic performance of PQ-FundusChain and the classical baselines is presented in Table 8.

### 7.1 On-chain storage

Because only a hash, a content identifier, short key material, the signature, and the access policy are stored per image, the on-chain record size is independent of image resolution and is fully determined by the field sizes of Table 2. Table 9 reports the computed per-image record for a single recipient and the incremental cost per additional recipient, alongside a classical RSA-3072/ECDSA-P-256 baseline.

End-to-end performance metrics, including cryptographic-path latency and smart-contract execution time, are reported in Table 10.

## 8 Discussion

**Figure 4:**
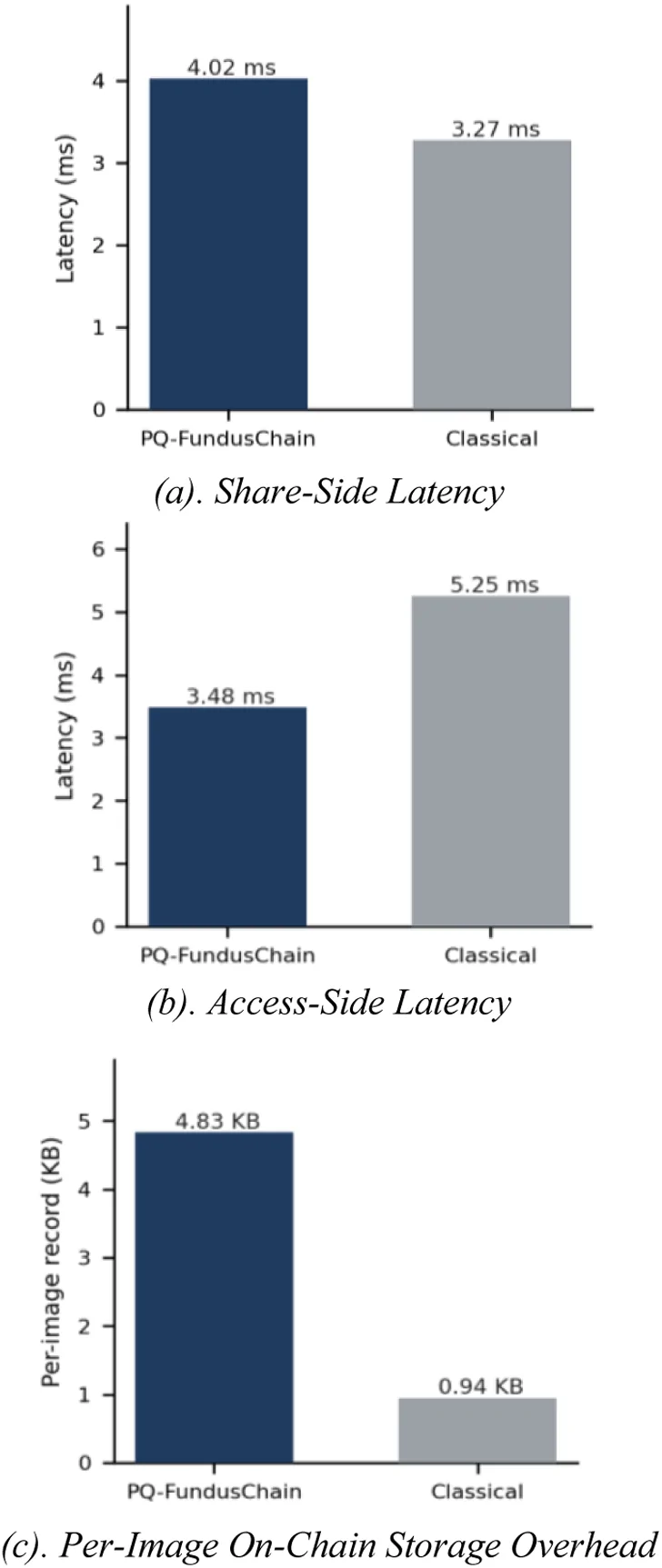
Performance comparison between PQ-FundusChain and a classical cryptographic baseline, showing **(a)** share-side latency, **(b)** access-side latency, and **(c)** per-image on-chain storage overhead. The results illustrate the trade-off between quantum-resistant security and system overhead, with PQ-FundusChain introducing less than 1 ms of additional share-side latency and approximately 5-fold higher on-chain storage, while reducing access-side latency relative to the classical baseline.

As shown in Table 8, the cost of quantum resistance remains modest on the cryptographic path, with ML-KEM-768 key generation, encapsulation, and decapsulation each completing in under 0.1 ms, vs. 176.6 ms for RSA-3072 key generation and 1.97 ms for RSA-OAEP decapsulation; ML-DSA-65 signing is the slowest postquantum operation at 0.76 ms. Aggregating per image for a single recipient, the share-side cryptographic and hashing cost is approximately 4.0 ms for PQ-FundusChain vs. 3.3 ms classically, an increase below 1 ms (Figure 4a), while the access-side cost is actually lower for the postquantum path (approximately 3.5 ms vs. 5.3 ms, Figure 4b), because ML-KEM decapsulation is much faster than RSA-OAEP decryption. SHA3-256 hashing of a 1 MB image (2.93 ms) dominates the cryptographic path, and together with the off-chain image transfer, it governs the wall-clock latency, whereas the symmetric encryption (approximately 0.26 ms) and the public-key operations contribute comparatively little. According to Table 9, the on-chain record is approximately 4.8 KB per image for a single recipient vs. 0.94 KB classically (Figure 4c), an overhead nearly 5-fold dominated by the ML-DSA signature and ML-KEM ciphertext and independent of image resolution. Despite the relative increase, the absolute per-image on-chain record stays at roughly 4.8 KB because the image data themselves are held off-chain, and hence, the ledger never grows with image size. In clinical terms, the submillisecond cryptographic overhead added per image is negligible against the time to transmit a fundus image across a referral network and against the minutes-to-hours timescale of teleophthalmology grading and referral, and hence, quantum-resistant protection imposes no perceptible burden on the screening workflow. The risk-aware layer adds only the policy parameters (approximately 160 bytes) and constant-time score arithmetic, and thus, its cost is negligible. The design is crypto-agile: ML-KEM and ML-DSA parameters can be raised to Category 5 for CNSA 2.0, and the symmetric primitive can be replaced by a region-aware image cipher, without changing the protocols. We also report a measured blockchain metric: Table 10 shows that both AccessGrant and PolicyUpdate execute in approximately 0.25 ms (median 0.24 ms, standard deviation 0.04 ms), measured by running the chaincode logic with the same primitives over 3,000 iterations on the Intel Xeon 2.80 GHz processor. The manuscript reports approximately 0.25 ms execution time and approximately 4,000 transactions/s as compute-bound validation throughput on a single endorsing peer and is independent of the consensus configuration. Dividing by the per-request cost gives a compute-bound validation throughput of approximately 4,000 transactions per second on a single endorsing peer, excluding ordering and consensus. Ledger throughput, commit latency, and block confirmation time additionally depend on ordering and consensus in a deployed consortium network. We do not report modeled values for these and identify their measurement on a deployed Hyperledger Fabric or equivalent network as the principal remaining validation step.

**Figure 5:**
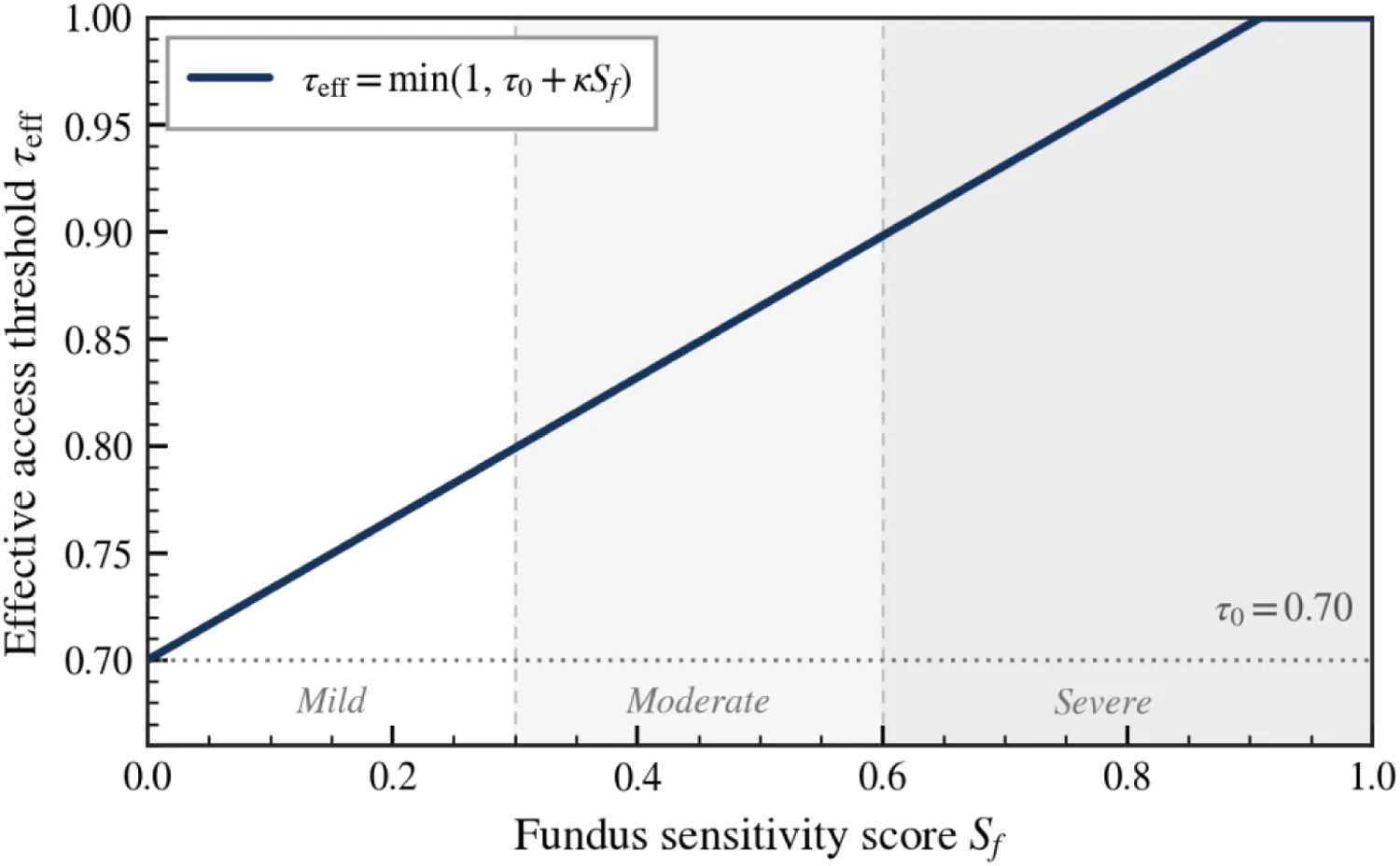
Sensitivity-adaptive access threshold in PQ-FundusChain.

Figure 5 visualizes the adaptive authorization mechanism: as the fundus sensitivity score $S_{f}$ rises, the effective access threshold $\tau_{eff}$ increases, and therefore, more sensitive images require a higher requester risk score before a key is released.

Table 11 consolidates the security analysis of Section 5 into a single reviewer-facing checklist, listing each objective, the mechanism that provides it, and whether the property is achieved.

**Table 11:** Security objective validation: each goal, its supporting mechanism, and whether it is achieved.

Security goal	Mechanism	Achieved
Confidentiality	AES-256-GCM + ML-KEM (FIPS 203)	✓
Integrity	SHA3-256 anchor+AES-GCM tag	✓
Authentication	ML-DSA (FIPS 204)	✓
Non-repudiation	ML-DSA signatures + immutable ledger	✓
Postquantum confidentiality (HNDL-resistant)	ML-KEM encapsulation, 256-bit keys	✓
Access control	Attribute policy + risk score R_u ≥ τ_eff	✓
Auditability	Consortium ledger + audit contract	✓
Revocation	Access/policy smart contract	✓
Availability	Decentralized storage + multipeer consensus	✓

## 9 Limitations

First, postquantum keys, ciphertexts, and signatures are larger than their classical counterparts, increasing transport and on-chain overhead. The computed analysis quantifies this as a roughly 5-fold larger on-chain record per image, whose effect at deployment scale should be evaluated on large consortium networks. Second, the risk-aware access-control model is evaluated through simulation: the trust, access-history, and location scores were generated from representative behavior profiles (Figure 3) rather than from operational teleophthalmology logs, and therefore, its weights, threshold, and scoring functions still require calibration and validation against real request traffic, and poorly chosen parameters could over- or under-restrict access. Third, the experiments use deidentified public images, and hence, end-to-end binding of each image to a live patient record remains to be validated. Fourth, attribute revocation propagates only at the speed of ledger commit, and therefore, a key already delivered before revocation cannot be recalled. Per-image keys and short grant windows limit the resulting exposure. Fifth, the security analysis is based on cryptographic reasoning and threat modeling and has not yet undergone formal symbolic verification. Finally, the framework has been evaluated in a controlled experimental environment rather than a production consortium network, and therefore, network-level performance characteristics were not measured.

## 10 Conclusion and future work

We proposed the PQ-FundusChain, a consortium-blockchain framework for secure teleophthalmology image sharing that resists both classical and quantum adversaries. It pairs postquantum security (ML-KEM key encapsulation and ML-DSA signatures) with blockchain-based sharing that keeps ciphertext off-chain, while anchoring hashes and attribute policies on-chain, and it gates key release through a risk-aware access-control model over trust, access-history, and location scores. Its fundus-specific adaptation, a clinically grounded sensitivity score that raises the authorization bar for the most diagnostically and privacy-sensitive images, distinguishes the framework from generic medical-image sharing schemes. Quantitatively, the evaluation showed a share-side cryptographic and hashing cost of approximately 4.02 ms per image, an access-side cost of approximately 3.48 ms, an on-peer smart-contract execution of approximately 0.25 ms for both AccessGrant and PolicyUpdate, and a per-image on-chain record of approximately 4.83 KB that is independent of image resolution, and therefore, quantum resistance is obtained for under 1 ms of additional share-side latency.

Future research will center around the validation of the risk-aware access-control model utilizing real teleophthalmology access logs and the implementation of pilot deployments within clinical screening programs. Other approaches could involve the use of formal protocol verification tools like ProVerif or Tamarin, testing on multipeer consortium networks, the merging of the model with electronic health-record systems, and the study of region-aware image encryption methods for the enhancement of privacy protection. A further direction is extending the risk-aware access-control model to capture time-varying and spatiotemporal patterns in access requests and trust evolution across the consortium network. Non-standard finite difference and fractional-order formulations offer a candidate numerical framework for such an extension once the underlying dynamics are specified.

## Data Availability

The Messidor-2 dataset used in this study is publicly available from ADCIS. Requests for access can be made through the official website: https://www.adcis.net/en/third-party/messidor2/. Access is subject to the dataset provider's terms of use. No accession number is available.
